# A Study on the Possible Link of Brucellosis to Increased Stillbirths in the Maltese Islands from 1919 to 1954

**DOI:** 10.1002/puh2.203

**Published:** 2024-06-21

**Authors:** Lianne Tripp, Larry A. Sawchuk, Mahinda Samarakoon

**Affiliations:** ^1^ Department of Anthropology Trent University Peterborough Ontario Canada; ^2^ Department of Anthropology University of Toronto Scarborough Toronto Ontario Canada; ^3^ Department of Computer and Mathematical Sciences University of Toronto Scarborough Toronto Ontario Canada

**Keywords:** *Brucella melitensis*, brucellosis, fetal loss, foodborne transmission, goats, male stillbirths, Malta

## Abstract

**Background:**

Human brucellosis, otherwise known as undulant fever, is one of the most widespread zoonotic diseases in the world. Even though 9%–15% of stillbirths are known to be caused by infectious diseases, the study of the link between human *Brucella melitensis* and the termination of births in humans is a topic that has received little attention. This study examines if there was an association between infection of undulant fever, an endemic zoonotic disease in the Maltese Islands from 1919 until 1954, and reproductive loss through stillbirths.

**Methods:**

A univariate descriptive analysis was used to show the temporal trend of undulant fever time, as well as the age and sex distribution. Time series analysis was used to assess the relationship between time (months) and undulant fever cases with stillbirth proportions.

**Results:**

On the island of Gozo, the majority of undulant fever cases for both males and females occurred in their reproductive period between 15 and 45 years of age. Based on regression analysis, undulant fever had a statistically significant effect on the stillbirth rate for males (*t* = 2.8986, *p* = 0.0039). The effect of undulant fever on stillbirths was not significant for females (*p* = 0.9103).

**Conclusion:**

This paper highlights the importance of undulant fever as having implications for the health burden in pregnant women and potential fetal loss through stillbirths in the contemporary context.

## Graphical abstract

In Malta, where there has been a long history of endemic brucellosis, we found a relationship between undulant fever infections and male stillbirths; this relationship has been largely ignored in the literature. This study points to health implications for locations where undulant fever is endemic today.

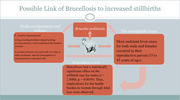

## 1 Introduction

The reemergence of undulant fever in Central Asia and the Middle East [1, 2, 3] is in large measure because of the change in epidemiology [4], the role of animal husbandry tradition, and the culture of raw milk consumption. These factors are also major reasons for the continued persistence of the disease in many countries around the world [5, 6].

One health implication of human brucellosis (*Brucella melitensis*), the most common and virulent species of undulant fever, is the potential role of the bacteria in inducing fetal loss. The etiologies of abortions and stillbirths are complex and can be attributed to either non‐infectious causes (e.g., congenital anomalies, placental insufficiency, placental abruption, and asphyxia) or to infectious agents [7]. Approximately 9%–15% percent of stillbirths are caused by infectious diseases and, in particular, infections early in pregnancy [8]. Economic factors can influence the role of infectious diseases and stillbirths. In middle‐ and low‐income countries, 50% or more of stillbirths can be attributed to infections, whereas, in high‐income countries, only 10%–25% of maternal or fetal infections can account for stillbirths [7, 9]. Specific infectious agents linked to stillbirths include syphilis, malaria, toxoplasmosis, parvovirus B‐19, chorioamnionitis, and *Listeria monocytogenes*. Less definitive links to stillbirths include the genital mycoplasmas, *Chlamydia trachomatis*, HIV, and group B streptococci among others [7, 8].

It has been well documented that animal strains of brucellosis cause abortions of fetuses and “reproductive failure,” in cows, dogs, goats, horses, pigs, sheep, and even camels [10, 11, 12]. *B. melitensis* can be transmitted to humans from goats or sheep through consumption of unpasteurized milk and cheese; through close contact with animals (i.e., hunting, milking, or caring for the sick); through meat processing and meat consumption; or through inhalation or airborne of the pathogen. Rarely, the bacteria can be transmitted from person‐to‐person either via sexual contact or airborne transmission.

Few studies have examined the relationship of human stillbirths and *B. melitensis*, especially at the cohort or population level [13, 14]. The majority of studies are case reports of stillbirth and abortions that are limited in scope and sample size [9, 15, 16]. These studies, along with clinical studies on brucella and abortion in placentas [17, 18], point to the strong possibility that brucellosis is a risk factor for adverse health outcome to the human fetus.

The Maltese Islands present a rare opportunity for a population‐based inquiry into the potential adverse effects of undulant fever on fertility through fetal loss. The advantages of the study site include the following population‐based attributes: Undulant fever (Malta or Mediterranean Fever) was a notifiable disease since the early 20th century; it remained endemic throughout the study period; monthly statistics of births and stillbirths by sex were published since 1900; pregnant women were at continuous and at high exposure to undulant fever because of high fertility rates owing to their adherence to strict Catholic precepts [19, 20]. Throughout the study period, the health care infrastructure was rudimentary along with little available prenatal care for expectant mothers, which put the vast majority of women at risk for fetal loss [19, 21, 22]. It has been documented that the infant mortality rates in Malta exceeded 200 per 1000 live births [23]. This article explores the relationship between human *B. melitensis* and stillbirths, in the civilian population of Malta during the period 1919–1954.

## 2 Methods

### 2.1 Study Design, Setting, and Population

This population‐based retrospective epidemiological study was conducted using primary and secondary data on undulant fever cases, births, and stillbirths on the Maltese Islands of Gozo and Malta from April 1919 until June 1954. The study period begins after the 1918/19 influenza epidemic and ends at the last year of data on undulant fever that the authors extracted from the Annual Health Reports. The total population size during the study period was 224,859 in 1919 at the beginning of the study and 311, 849 people near the close of the study in 1949.

There are two stages of the study. The first stage of study was descriptive. The annual distribution of undulant fever cases for both islands was examined. The age and sex distribution of the undulant fever cases for Gozo was assessed to ascertain the potential of undulant fever to affect those in their reproductive prime, which then could contribute to fetal loss.

The second stage of analysis examined the relationship between the number of monthly undulant fever cases and stillbirth rates. Time series analysis was used to model the seasonal relationship as measured by months between the dependent variable, stillbirth proportions and the independent variables, time and undulant fever cases.

### 2.2 Study Variables and Data Source

The records, monthly births, stillbirths, and undulant fever case information, were photographed from archives in London and Malta. All information was digitized and stored on a secure server.

Monthly notifications of undulant fever cases were extracted from the Annual Health Reports on the Health of the Maltese Islands. The reports are housed at the National Archives of Malta (NAM), and The National Archives (TNA) in Kew, England. The few missing values were estimated by linear interpolation [24]. Age and sex notification information on undulant fever was only available for the sister smaller island of Gozo from the Health Office in Gozo; the records for Malta were unfortunately destroyed. Monthly numbers of births and stillbirths by sex from April 1919 to June 1954 were drawn from the Maltese Gazette that was published under the auspices of the Medical Officer of Health and was accessed at the NAM and TNA.

### 2.3 Data Analysis

In the descriptive analysis phase, the temporal trend of undulant fever cases from 1919 until 1954 was examined. The age and sex distribution of undulant fever cases on the island of Gozo from 1919 until 1940 was also explored.

**FIGURE 1:**
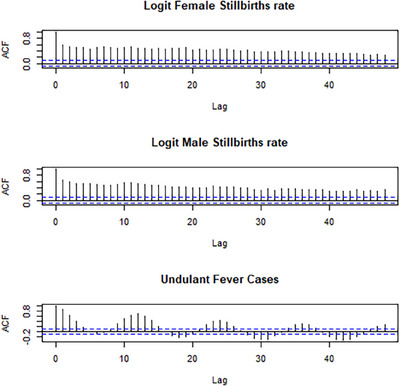
Time series plots of logit transformed monthly stillbirth prevalence for males and females, and for the independent variable of undulant fever cases, showing the autocorrelation function.

Time series analysis was used to model a relationship between the dependent variable and independent variables. These models assume the error terms to be independent. This assumption is frequently violated when applied to time series data. The regression model with integrated autoregressive moving average (ARIMA) errors allows for correlated errors. Consequently, in order to study the relationship between the number of undulant fever cases and the proportion of stillbirths, a regression model was used, which allows auto‐correlated errors and has an ARIMA process. A logit transformation on the proportion of stillbirths was used, in particular the logarithms of the odds of stillbirths (i.e., $\log(\frac{p}{1-p}))$, where *p* denotes the proportion of stillbirths as the dependent variable, and time (months) and undulant fever cases are the explanatory variables. Because the time series data showed significant autocorrelations and cross correlations at many lags indicating serial correlations in the time series for each sex (see Figure 1), a logistic regression model was fitted with the logit of the stillbirth rate as the dependent variable and the time (month), and the number of undulant cases as the independent variables. The models were tested for multicollinearity, based on variance inflation factors. They are all well below 10, the usual critical value, indicating that no serious multicollinearity exists between the independent variables. The best suited ARIMA models for the resulting residuals [25] were used as the process of generating the errors of the regression models [26, 27]. The residual plots for the models fitted did not indicate serious violations of the assumptions. The number of stillbirths and undulant fever recorded at the different months were random in nature and displayed an approximately normal distribution.

The graphs were created in Statistica [28], and the regression model was completed in R statistical software [27].

### 2.4 Ethical Considerations

Ethical approval was not required for this study because human subjects were not included in the study, and the data included only aggregated information that did not reveal any personnel identifiers.

## 3 Results

Over the study period, there were 161,228 male births and 148,729 female births across the two Maltese Islands. The total numbers of male and female stillbirths were 8011 and 4071, respectively.

**FIGURE 2:**
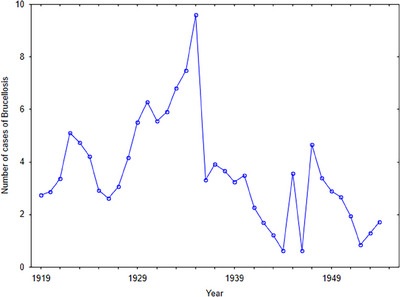
Undulant fever cases in the Maltese Islands, 1919–1954.

Undulant fever was endemic to the islands with periodic occurrences of major epidemics (see Figure 2). As there was no significant difference in the mean number of cases between Malta and Gozo, the undulant fever numbers for the two islands were pooled. The mean number of undulant fever cases was 3.611 per 1000 living over the study period.

**FIGURE 3:**
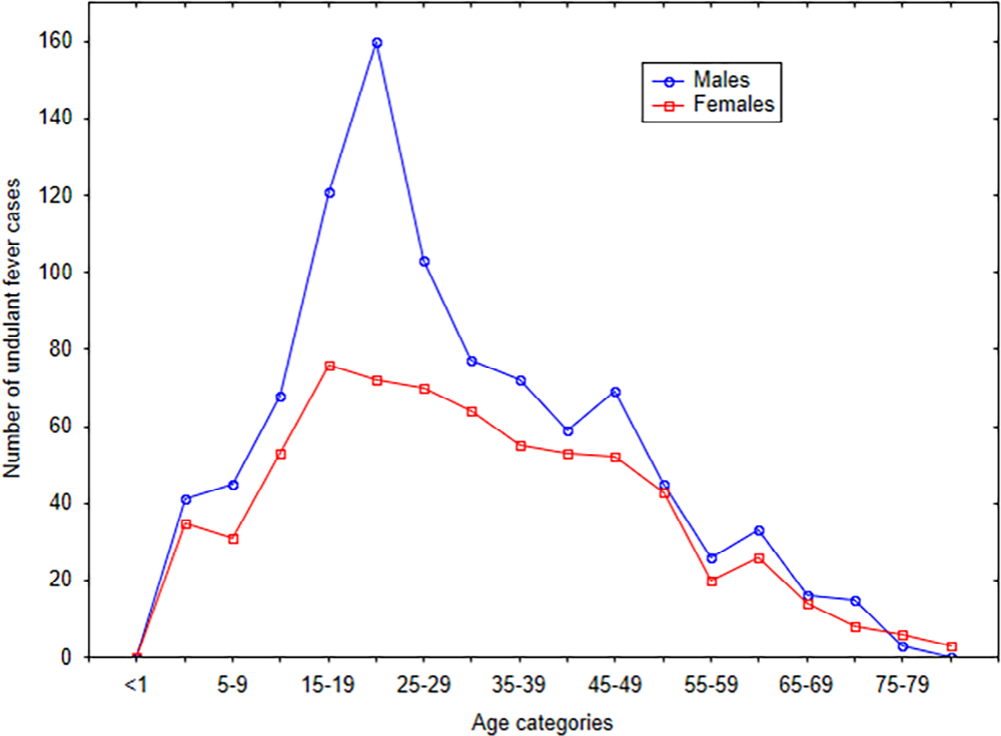
Undulant fever cases by sex in Gozo from 1919 to 1940.

Figure 3 shows cases of undulant fever by age and sex in Gozo and presumably reflective of that in Malta. The group most at risk of the disease were males (*n* = 961) with the most cases occurring in the age band of 20–24 years of age (see Figure 3). The peak age group for females (*n* = 684) was 15–19 years of age and the number of cases remained elevated throughout the reproductive years. Among males, 69.36% of cases occurred in that age group. For females, 71.22% of cases occurred in women aged 15–49 years.

The regression model showed that undulant fever had a statistically significant effect on the male stillbirth rate (*t* = 2.8986, *p* = 0.0039), but the effect of undulant fever on female stillbirths was not statistically significant (*p* = 0.9103).

## 4 Discussion

There was a significantly increased risk of male stillbirths associated with undulant fever after controlling for seasonality associated with birthing on the Maltese Islands from 1919 to 1954. Generally, the literature on sex‐based differences in human stillbirths indicates that male stillbirths are more common than female stillbirths [29, 30, 31]. This study shows that the male fetus was significantly more vulnerable under an insult from undulant fever than a female fetus. This study could only address this issue in broad terms. A plausible explanation as for why male stillbirths are more common is that pregnant mothers under adverse environmental conditions initiate different coping strategies according to the sex of the fetus [32]. Female fetal growth was reduced, whereas, in contrast, the male fetus continues to grow which can ultimately result in preterm delivery and stillbirth.

Undulant fever was endemic to the Maltese Islands because the goat was deeply rooted in the social fabric of Maltese culture and daily life. Topographically, Malta was considered to be a place of “poverty of the grazing land,” which gave scant opportunity for cattle breeding but favored the breeding of small ruminant such as goats [19]. The densely populated towns with short distances between villages, towns, and cities made the goat an ideal candidate for distributing milk to the customers doorsteps [33]. Without refrigeration, the majority of the population were compelled to rely on foods that were consumable on a daily basis.

Despite concerted advice from local medical men and colonial authorities, the consumption of drinking fresh unpasteurized goat milk as well as their milk byproducts went unheeded. The belief system was based on the conviction that boiling milk was not necessary because: (1) it ruined the quality and flavor of milk; (2) it was considered superfluous by the people because it was known that the Maltese goat did not suffer naturally from tuberculosis, which is why the precautions of boiling milk had been advocated abroad; but now great emphasis was laid on this simple yet effective measure; (3) It was thought that having the goat milked at the door meant that contamination or adulteration of the milk was not possible; (4) it did not align with the general public belief that a “normal beverage drawn straight from the familiar goat can be productive of a deadly fever”; and (5) it was simply unobtainable given that the poor simply did not have the means to boil milk [19].

The Gozo notification records show that both sexes were likely to contract undulant fever during their reproductive period. The fact that adults were more likely to fall ill with the disease is not surprising, considering that children and in particular infants were not encouraged to drink milk [34]. Consequently, consumption of fresh goat milk could have had the potential to affect fetal loss through stillbirths.

Today, undulant fever in humans does not appear to be a health concern on the island. Changes to how the disease was controlled, in Malta, began in 1957 with the complete prohibition of the sale of raw milk [35]. Nonetheless, cases continued into the 1980s. In 1988, health officials made a concerted effort to cull all infected goats and cows in Malta and Gozo. In 1995, there was an outbreak of 130 cases with one death after the consumption of cheeselets made with goats’ cheese [36]. Since then, there have not been cases of undulant fever except for the occasional case imported from abroad [37].

## 5 Conclusion

Undulant fever was endemic to the islands with an epidemic peak in the 1930s, and this was followed by a progressive decline until the 1980s with occasional sporadic cases. Based on the smaller sister island of Gozo, cases were found primarily in young adult males and, to a lesser extent, in females in their reproductive prime. After adjusting for seasonality in births by month, undulant fever had a significant effect on fetal loss through stillbirths of males, but not females. Although the results of this zoonotic disease are confined to historic Malta, the fact that undulant fever continues to be a health and economic burden in other countries suggests that other researchers should study the relationship of undulant fever and stillbirths in the modern context.

## Author Contributions

**Lianne Tripp**: conceptualization, funding acquisition, writing–original draft, data curation, supervision, writing–review and editing, investigation, formal analysis, methodology, project administration, resources, visualization. **Larry A. Sawchuk**: data curation, conceptualization, investigation, funding acquisition, writing–original draft, software, writing–review and editing, visualization, methodology, validation, formal analysis, resources. **Mahinda Samarakoon**: methodology, validation, formal analysis, software, writing–review and editing, investigation, writing–original draft.

## Conflicts of Interest

None of the authors have conflicts of interest to disclose.

## Data Availability

Data is available upon request.
